# Effects of Aqueous Leaf Extract of *Lawsonia inermis* on Aluminum-induced Oxidative Stress and Adult Wistar Rat Pituitary Gland Histology

**DOI:** 10.5935/1518-0557.20190024

**Published:** 2019

**Authors:** Toluwase Solomon Olawuyi, Kolade Busuyi Akinola, Sunday Aderemi Adelakun, Babatunde Samson Ogunlade, Grace Temitope Akingbade

**Affiliations:** 1 Department of Anatomy, School of Health and Health Technology, Federal University of Technology, Akure (FUTA), Nigeria

**Keywords:** adenohypophysis, neurohypophysis, asthenospermia, teratospermia

## Abstract

**Objectives:**

The aim of this study was to investigate the antioxidant effect of aqueous *Lawsonia inermis* leaf extract on aluminum-induced oxidative stress and the histology of the pituitary gland of adult Wistar rats.

**Methods:**

Thirty-five adult male Wistar rats weighing between 100-196g and 15 mice of the same weight range were included in the study. *Lawsonia inermis* extracts and aluminum chloride (AlCl_3_) were administered for a period of three weeks to five rats per group. The subjects in Group 1 (control) were given pellets and distilled water. Group 2 received 60mg/kg/d of aqueous extract of *Lawsonia inermis*. Group 3 was given 0.5mg/kg/d of AlCl_3_. Group 4 was administered 0.5mg/kg/d of AlCl_3_ and 60mg/kg/d of aqueous *Lawsonia inermis* extract orally. Group 5 received 0.5mg/kg/d of AlCl_3_ and 75mg/kg/d of aqueous *Lawsonia inermis* extract orally. Group 6 was given 0.5mg/kg/d of AlCl_3_ and 100mg/kg/d of aqueous *Lawsonia inermis* extract orally. Group 7 was administered 0.5mg/k/d of AlCl_3_ and 5mg/Kg/d ascorbic acid in distilled water orally. Twenty-four hours after the last administration, the animals were weighed, sedated with chloroform, and had their pituitary glands located, removed, and weighed on an electronic analytical balance.

**Results:**

Decreased cell counts were observed in the pituitary gland micrographs of the Wistar rats given 0.5mg of aluminum chloride, whereas the Wistar rats given 0.5mg of aluminum chloride and varying doses of *Lawsonia inermis* had increased dose-dependent cell counts.

**Conclusion:**

Aqeuous *Lawsonia Inermis* leaf extract increased the cell counts of the pituitary glands of adult male Wistar rats, in addition to alleviating aluminum-induced oxidative stress.

## INTRODUCTION

The pituitary gland, often called the "master gland" of the body, physiologically regulates the endocrine function of several other glands and their associated activities. The pituitary gland can be functionally divided into two parts: the posterior (neurohypophysis) and the anterior (adenohypophysis). Lower animal species possess a third part that is not present in humans, known as pars intermedia. Developmentally, the anterior pituitary and the posterior pituitary have different origins. The anterior pituitary develops from Rathke's pouch, an invagination from pharyngeal epithelium, while the posterior pituitary develops from a neural tissue outgrowth from the hypothalamus (Guyton & Hall, 2006).

The anterior pituitary gland contains numerous basophil cells. The counts of acidophil cells (arranged in cords) were lower than the basophil and chromophobe cell counts. Acidophil cells may occur in two different forms based on their size and shape. Type 1 cells are found near the sinusoids and have irregular shapes, while Type 11 cells are round and have coarse chromatin granules. Basophil cells are larger and occur in greater number than acidophil cells. They are categorized into two types of different shapes and sizes. Basophil cells stain magenta-bluish (Young *et al.,* 2006). Chromophobe cells are round and are the largest of the group. These cells are located centrally and have dark nuclei, clear nucleoli, and granular cytoplasm (Young *et al.,* 2006).

The anterior pituitary secretes six important peptide hormones, while the posterior pituitary secretes two peptide hormones. Male reproductive function is regulated by the follicle stimulating hormone (FSH), which stimulates the spermatogenic epithelium, and the luteinizing hormone (LH), which stimulates the production of testosterone by Leydig cells in interstitial tissue. Follicle stimulating hormone (FSH) is found in humans and other animals. It is synthesized and secreted by gonadotropic cells of the anterior pituitary gland. FSH regulates the development, growth, pubertal maturation, and reproductive processes of the body. FSH and Luteinizing hormone (LH) act synergistically in reproduction (Olawuyi *et al.,* 2017).

For over 9,000 years, Henna - or *Lawsonia inermis* - has been used to draw skin tattoos. Apart from its cosmetic uses, *Lawsonia inermis* has been used as a hair-coloring agent in many parts of the world (Sridhar *et al.,* 2016). *Lawsonia inermis* is a medicinal plant used to treat gonorrhea, herpes infection, rheumatic conditions, wounds, in addition to having anti-neuralgic and anti-diabetic properties (Syamsudin & Winarno, 2008; Mohammad, 2016). Some of the phenolic compounds found in the plant can be used to design effective drugs against heavy metal poisoning (Towolawi *et al.,* 2010).

In the course of their lives, humans are exposed to potentially harmful environmental pollutants such as aluminum (Mohammadirad & Mohammad, 2011). According to Ghalberg & Brodas (1981), aluminum may pathologically alter the testes and induce testicular atrophy. The potential effects of aluminum poisoning include asthenospermia, hypospermia, teratospermia, and decreased sperm count. The American Association of Poison Control Centers reported 813 single exposures to aluminum in 2013, with seven moderate outcomes and no major outcomes or deaths (Olawuyi *et al.,* 2017; Bell & Thomas, 1980; Balasubramanyam *et al.,* 2009). Advances in nanotechnology have led to the exposure of humans to aluminum in engineered nanomaterials (NMs) that may potentially induce genomic changes.

## MATERIALS AND METHODS

Aluminum chloride and ascorbic acid were procured from the Mich-Deson Hospital Equipment store, Upper Taiwo, Ilorin. Staining procedures were carried out in the Pathology Department, University Teaching Hospital Ilorin, Nigeria.

### Preparation of Extracts

Plant samples were procured in Isanlu-Isin, Kwara State, Nigeria and had their identities confirmed identified and assigned herbarium number **UPH/P/114** by the Taxonomist of the Department of Plant Science and Biotechnology, University of Port-Harcourt, Rivers State, Nigeria. The Research Ethics Committee of the same institution approved the study on February 25, 2016, and gave it reference number **UPH/CEREMAD/REC/04**. The plant leaves were washed with water, cut into pieces, and dried in a cool environment. The dried plant leaves were then pulverized into a coarse powder with the aid of a grinding machine. The filtrate was concentrated using a rotary evaporator (Buchi) and further concentrated to dryness at 50ºC in an electric oven (GallenKamp). After drying, the samples were stored in a refrigerator at 4ºC until the time they were used.

### Acute Toxicity Testing (LD_50_)

Fifteen mice were used in the tests to determine the safe and lethal dosages of the extract. The animals were split into five groups, each with three individuals. The acute toxicity of the aqueous extract of *Lawsonia inermis* leaves was assessed based on the LD_50_ calculation, with a limit dose of 1000mg/kg of body weight of the extract administered orally (three animals per group) (OECD-OCDE 425 Guide). The mice given the extract orally showed dose-dependent signs of toxicity, which ranged from lack of appetite, depression, immobility, and respiratory distress to death. The LD_50_ for the *Lawsonia inermis* extract was 0.75g, while the safe dose was 0.1g/Kg b.w.

### Determination of the Dosage of the Extract to Administer

The choice of dosage was based on the acute toxicity test (LD_50_) cited above, in which the safe dose of *Lawsonia inermis*is was set at 0.1g/Kg or 100mg/Kg body weight. The highest dose was 100mg/Kg, the medium dose was 75mg/Kg, and the lowest dose was 60mg/Kg.

### Breeding of the Animals

Thirty-five adult male Wistar rats and fifteen mice were included in the study. The subjects weighed between 100g and 196g. After procurement, the rats were housed in cages (made with wood, wire gauze, and mesh) kept at room temperature and subject to cycles of natural light and darkness at the animal house of the Faculty of Basic Medical Sciences, University of Ilorin. The floor of the cages was made of wood to make it comfortable for the rats and was covered with sawdust to provide a soft floor for the rats and to make cleaning of the cage convenient when littered. They were fed with pellets purchased from approved stores by the University of Ilorin and given water *ad libitum*. They were grouped and left to acclimatize for two weeks before the start of the study.

### Grouping

A total of 35 animals were included in the study. They were grouped into one control and six case groups with consideration to size variations. Using a feeding tube (size-6), distilled water and portions of *Lawsonia inermis* extract were administered to the control and case animals respectively for a period of three weeks.

Group 1 (control): (n=5): Given rat pellets and distilled water.

Group 2: (n=5): Given 60mg/kg/d of extract of *Lawsonia inermis* and pellets.

Group 3: (n=5): Given 0.5mg/kg/d of aluminum chloride in distilled water and pellets.

Group 4: (n=5): Given 0.5mg/kg/d of aluminum chloride and 60mg/kg/d (low dose) of *Lawsonia inermis* in distilled water orally.

Group 5: (n=5): Given 0.5mg/kg/d of aluminum chloride and 75mg/kg/d (medium dose) of *Lawsonia inermis* orally.

Group 6: (n=5): Given 0.5mg/kg/d of aluminum chloride and 100mg/kg/d (high dose) of *Lawsonia inermis* in distilled water orally.

Group 7: (n=5): Given 0.5mg/k/d of aluminum chloride and 5mg/Kg/d of ascorbic acid in distilled water orally.

### Animal Sacrifice and Sample Collection

Twenty-four hours after the last administration, the animals were weighed and thereafter sacrificed with chloroform as a sedative. Their pituitary glands were located and removed.

### Histology and Histochemistry Analyses

Tissue specimens were taken from the pituitary glands of subjects from each of the seven groups and were fixed in formaldehyde and calcium chloride for 24 hours. Then each specimen was sliced into small slabs (3-5mm thick) and further fixed in a change of the same fixative for another 15 hours. The fixed tissue specimens were trimmed and washed in tap water for 12 hours. An alcohol series (methyl, ethyl, and absolute) was used to dehydrate the tissue specimens. The tissue specimens were cleared in xylene and embedded in paraffin. The paraffin blocks were sectioned in 5-micron slices on a rotary microtome. The obtained tissue sections were collected on glass slides and stained with Hematoxylin and Eosin, Periodic Acid-Schiff, and Orange G.

## RESULTS

### Histological Observation

**Plate 1:**
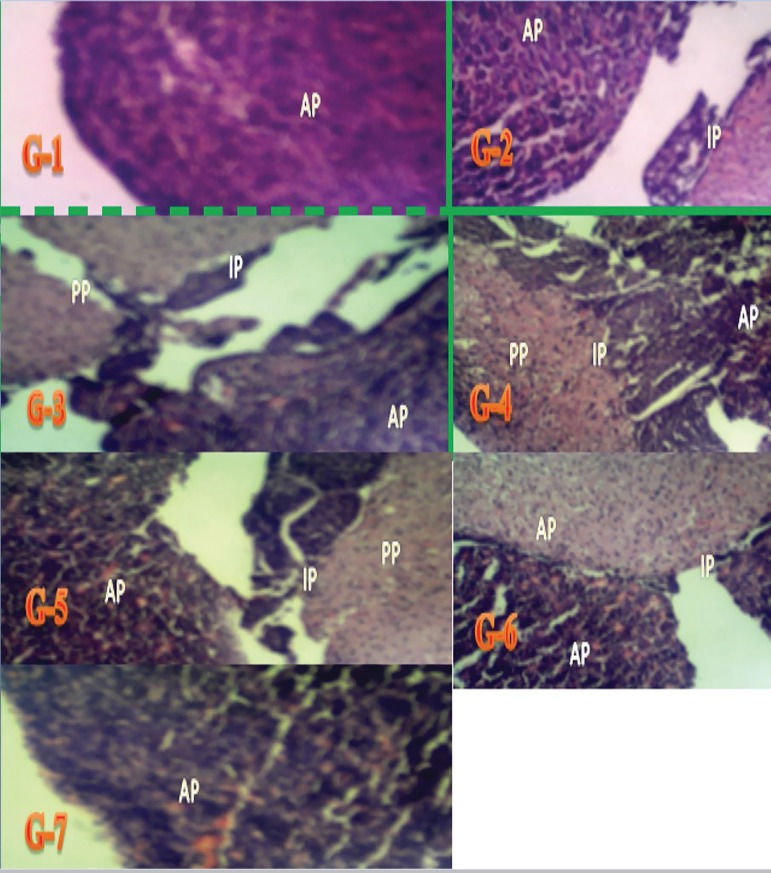
Representative pituitary gland micrographs of Wistar rats from Groups 1-7 showing the anterior pituitary gland (AP), the posterior pituitary gland (PP), and the pars intermidia (IP). Staining: H&E. Magnification x100.

**Plate 2:**
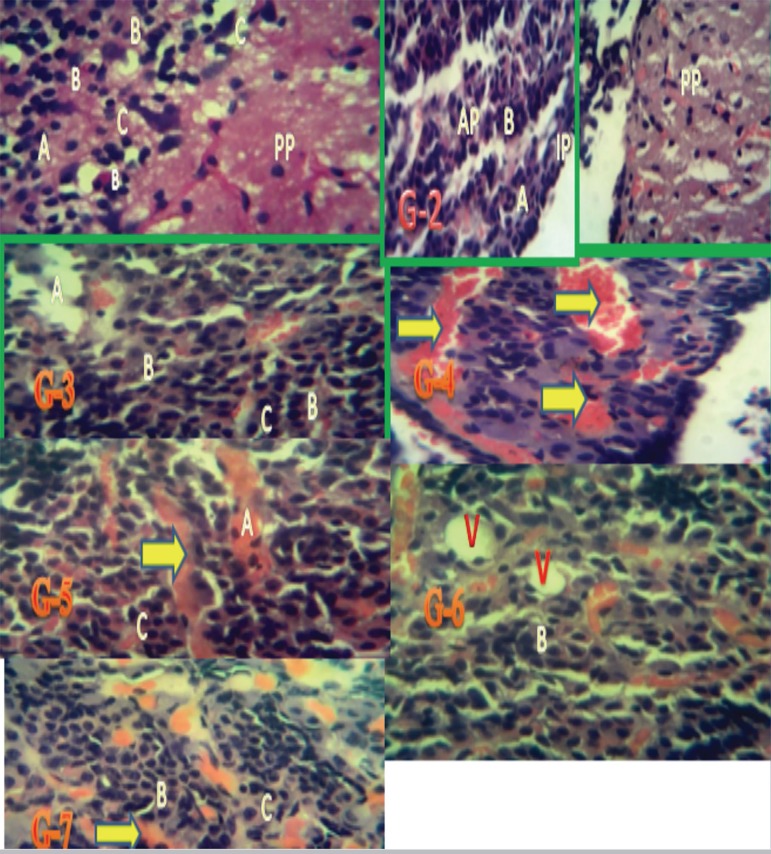
Representative pituitary gland micrographs of Wistar rats from Groups 1-7 showing areas of degenerative change. The posterior pituitary (PP), the anterior pituitary gland (AP) with increase basophil (B) and acidophil (A) cell counts and few chromophobe cells (C). Staining: H&E. Magnification: x400.

Effect of *Lawsonia inermis* leaf extract and aluminum chloride on the histology of the pituitary glands (H&E/PAS/Orange G) of the groups are shown Plates 1 and 2 below.

### Plates

- **Group 1:** Plates 1 and 2 show representative pituitary gland micrographs of Wistar rats from the control group with a normal pars distalis containing normal chromophil and chromophobe cells. Stained with H&E/PAS/Orange G; magnification x100 & x400.
- **Group 2:** Plates 1 and 2 show representative pituitary gland micrographs of Wistar rats given 60mg/kg of extract of *Lawsonia inermis* with normal pars distalis, pas intermedia, and pas nervosa. Stained with H&E/PAS/Orange G; magnification x100 & x400.
- **Group 3:** Plates 1 and 2 show representative pituitary gland micrographs of Wistar rats given 0.5mg of aluminum chloride with a pars distalis with decreased counts of chromophil and chromophobe cells, pas intermedia and pas nervosa. Stained with H&E/PAS/Orange G; magnification x100 & x400.
- **Group 4:** Plates 1 and 2 show representative pituitary gland micrographs of Wistar rats given 0.5mg of aluminum chloride and 60mg/kg of *Lawsonia inermis* with a pars distalis with reddish-yellow large stained cells, decreased number of chromophil and chromophobe cells, pas intermedia and pas nervosa. Stained with H&E/ PAS/Orange G; magnification x100 & x400.
- **Group 5:** Plates 1 and 2 show representative pituitary gland micrographs of Wistar rats given 0.5mg of aluminum chloride and 75mg/kg of *Lawsonia inermis* with the pars distalis with reddish-yellow large stained cells and a moderately increased number of chromophil and chromophobe cells, pas intermedia and pas nervosa. Stained with H&E/PAS/Orange G; magnification x400.
- **Group 6:** Plates 1 and 2 show representative pituitary gland micrographs of Wistar rats given 0.5mg of aluminum chloride and 100mg/kg of *Lawsonia inermis* with a pars distalis with reddish-yellow large stained cells and increased counts of chromophil and chromophobe cells, pas intermedia and pas nervosa. Stained with H&E/ PAS/Orange G; magnification x400.
- **Group 7:** Plates 1 and 2 show representative pituitary gland micrographs of Wistar rats given 0.5mg of aluminum chloride and 5mg/Kg of ascorbic acid with a pars distalis with reddish-yellow large stained cells and increased counts of chromophil and chromophobe cells, pas intermedia and pas nervosa. Stained with H&E/PAS/Orange G; magnification x400.

## DISCUSSION

Physiologically, the pituitary gland is made of two parts: the anterior pituitary (the adenohypophysis) and the posterior pituitary (neurohypophysis). Between these two parts lies a relatively small avascular zone called the pars intermedia. The hypothalamic-pituitary-testicular (HPT) axis is a hormonal axis of communication between the hypothalamus, the pituitary, and the testes that regulates male reproductive function.

In this study, the subjects in the control group had normal pituitary gland morphology (Plates 1 and 2) with a more cellular anterior pituitary gland and a posterior pituitary gland separated by the pars intermedia. The anterior pituitary gland contained clusters of cells grouped according to their affinities to dyes, with acidophil cells stained orange-brown, basophil cells stained dark-blue, and chromophobe cells stained light blue.

According to Plates 1 and 2, the histological architecture of the pituitary glands of the rats given 60mg/kg of aqueous extract of *Lawsonia inermis* could be easily distinguished by an anterior pituitary with numerous cells including basophil (gonadotropin secreting cells), acidophil, and chromophobe cells. Basophil cell counts were higher than the counts of acidophil and chromophobe cells, and were markedly increased when compared to the other groups. In addition, basophils and eosinophils that had recently undergone degranulation or were in the process of active hormone synthesis appeared as chromophobe cells.

In this study, the anterior pituitary of the rats given 0.5mg of aluminum chloride alone had few basophil, acidophil, and chromophobe cells. Numerous areas with degenerative changes were also present. Secretory cells had generally decreased counts when compared to the other groups, as previously described by Olawuyi *et al*. (2017), leading to dramatic decreases on the level of gonadotropic hormones (FSH and LH), as also described by Mohammadirad & Mohammad (2011), in a study that found that aluminum toxicity increases oxygen free radical levels and lipid peroxidation, which in turn decreases glutathione (GSH) levels. Kara *et al*. (2005) reported that glutathione as a substrate directly reacts with free radicals in enzymatic antioxidant reactions, thus protecting cells against oxidative stress. According to Jensen *et al*. (2006), metals negatively affect the neuroendocrine system and the Leydig cells, thus potentially affecting the secretion of androgen. In our study, a marked drop was noted in the levels of FSH and LH.

When aluminum chloride and different doses of *Lawsonia inermis* aqueous leaf extract (60mg/Kg, 75mg/kg and 100mg/Kg) were concomitantly given, the counts of basophil cells surpassed the counts of acidophil and chromophobe cells and were markedly higher than the counts seen in the group given the low-dose protocol. The effect of *Lawsonia inermis* extract was found to be dose-dependent. Doses of up to 100mg/Kg improved cell counts, while doses greater than 100mg/Kg possibly had a synergistic effect with aluminum to further decrease cell counts. The pars distalis had few areas with destructive changes and vacuolation areas (Plates 1 and 2). Moreover, as the dose increased the areas with destructive changes and vacuolation areas were significantly reduced. This finding is in agreement with Towolawi *et al.* (2010), in which an antioxidant role against heavy metal toxicity was described.

Lastly, the histology and morphology of the pituitary glands of the rats given 0.5mg of aluminum chloride and 5mg/Kg of ascorbic acid (Plates 1 and 2) revealed that the anterior pituitary gland could be well distinguished from the posterior pituitary gland by the elevated cell counts slightly greater than the counts seen in the subjects on the high-dose protocol (100mg/Kg of aqueous extract of *Lawsonia inermis*) with aluminum chloride for basophil (gonadotropin secreting cells), acidophil, and chromophobe cells. Basophil cell counts were greater than the counts of acidophil and chromophobe cells. There were few areas of destructive change related to aluminum intake. Periodic acid-Schiff (PAS) and Orange G staining are used to stain neuroendocrine cells. The numerous basophil cells present in the anterior pituitary stained magenta; acidophil cells stained yellow; the few chromophobe cells stained pale blue-grey; nuclei stained dark blue; and red blood cells stained yellow.

Lastly, acidophil cell counts were lower than the counts of basophil and chromophobe cells and are found as cords. Basophil cells are larger than acidophil cells and appear in clusters. Chromophobe cells have centrally-located nuclei, are round in shape, have granule-free cytoplasm, and are larger than other cells. In this study, Hematoxylin and Eosin (H&E) (Plates 1 and 2) and Periodic acid-Schiff (PAS)/Orange G staining (Plates 1 and 2) revealed similar increases or decreases in cell counts in the aluminum chloride and aqueous extract administration protocol. Similar pathological structures were also shown. The cells were more easily distinguished based on the colors they acquired after staining.

## CONCLUSION

This study showed the effects of aqueous *Lawsonia Inermis* leaf extract in increasing the cell counts of the pituitary glands of adult male Wistar rats and alleviating the oxidative stress induced by aluminum poisoning.
